# Association Between Admission Random Blood Sugar and Cardiorenal Syndrome Type 1 in Acute Decompensated Heart Failure

**DOI:** 10.3390/jcm15218687

**Published:** 2026-11-07

**Authors:** Elbadri I. Abdelgadir, Khamess O. Khamees, Lulwah Al Turki, Abdullah Al Thani, Mohammad A. Ellouly, Rami I. Abulikailik, Waleed G. Gado, Salem Alanazi, Ahmed Bakillah

**Affiliations:** 1 King Abdulaziz Hospital (KAH), Ministry of National Guard–Health Affairs (MNGHA), Al-Ahsa 36428, Saudi Arabia; khamisko@mngha.med.sa (K.O.K.); alturkilu@mngha.med.sa (L.A.T.); althaniab@mngha.med.sa (A.A.T.); elloulymo@mngha.med.sa (M.A.E.); abulikailikra@mngha.med.sa (R.I.A.); gadowa@mngha.med.sa (W.G.G.); alanazisa31@mngha.med.sa (S.A.); 2 King Abdullah International Medical Research Center (KAIMRC), Al-Ahsa 36428, Saudi Arabia; bakillaha@kaimrc.med.sa; 3 Biomedical Research Core A, King Saud Bin Abdulaziz University for Health Sciences (KSAU-HS), Al-Ahsa 36428, Saudi Arabia

**Keywords:** acute decompensated heart failure, admission random blood sugar, cardiorenal syndrome type 1, stress hyperglycemia, renal dysfunction

## Abstract

**Background**: Cardiorenal syndrome type 1 (CRS1) is an important complication of acute decompensated heart failure (ADHF). Admission hyperglycemia may reflect chronic dysglycemia, acute stress physiology, or both, but its relationship to CRS1 remains incompletely characterized. The primary objective was to assess the association between admission RBS and CRS1 in patients with ADHF. Secondary objectives were to compare RBS by CRS1 status and to explore its relationships with renal function, diabetes, and length of hospitalization. **Methods**: The study included 314 records (non-CRS1, *n* = 153; CRS1, *n* = 161), and admission random blood sugar (RBS) was available in 312. Cluster-robust logistic regression accounted for repeated admissions within patients and evaluated the association between CRS1 status and continuous RBS, with sequential adjustment for age, sex, diabetes, hypertension, prior acute kidney injury (AKI), chronic kidney disease (CKD), chronic obstructive pulmonary disease (COPD), estimated glomerular filtration rate (eGFR), and glycated hemoglobin (HbA1c). Exploratory analyses evaluated RBS categories, diabetes interaction, RBS–eGFR correlation, discrimination, and hospitalization duration. **Results**: Admission RBS was higher in CRS1 than in non-CRS1 records (11.02 ± 6.07 vs. 9.74 ± 5.04 mmol/L; Welch *p* = 0.044; Mann–Whitney *p* = 0.049). Each 1 mmol/L RBS increase was associated with CRS1 in the unadjusted model (OR = 1.042, 95% CI = 1.004–1.082; *p* = 0.030), but not after adjustment for age, sex, and diabetes (OR = 1.034, 95% CI = 0.993–1.076; *p* = 0.103) or the expanded clinical model (OR = 1.049, 95% CI = 0.989–1.112; *p* = 0.111). Adding eGFR or HbA1c did not yield a significant association. RBS showed poor discrimination (AUC = 0.564, 95% level bootstrap CI = 0.501–0.627, 3000 resamples of 198 unique patients). Prolonged hospitalization (>7 days) was not associated with CRS1 or admission RBS. **Conclusions**: In this retrospective study, admission RBS was higher in ADHF records classified as CRS1; however, after adjusting for clinical risk factors, the association attenuated, and RBS showed limited discriminatory ability. No significant correlation between RBS and eGFR was observed within the CRS1 group. These findings do not establish a causal relationship between admission glycemia and CRS1, nor do they support using RBS alone to identify CRS1. The LOS findings should be interpreted cautiously because of substantial missing data and the potential for selection bias. Prospective studies incorporating standardized and longitudinal glycemic measures, HbA1c, and established cardiorenal markers are warranted to determine whether comprehensive glycemic assessment improves CRS1 risk stratification.

## 1. Introduction

Acute decompensated heart failure (ADHF) is characterized by worsening congestion, elevated cardiac filling pressures, altered systemic perfusion, and neurohormonal activation [1,2]. Acute kidney dysfunction occurring in this setting is clinically framed as cardiorenal syndrome type 1 (CRS1). Its pathophysiology is multifactorial and involves renal venous congestion, impaired effective perfusion, neurohormonal activation, inflammation, endothelial dysfunction, and treatment-related hemodynamic changes [3,4,5,6]. Importantly, renal dysfunction in ADHF cannot be attributed solely to reduced cardiac output or arterial underperfusion [7]. Increased central venous pressure and renal venous congestion may impair the renal perfusion gradient, elevate interstitial pressure, and contribute to declining kidney function even when systemic blood pressure is maintained. These interacting hemodynamic and nonhemodynamic mechanisms underscore the complexity of identifying patients at risk of CRS1 with a single circulating biomarker [8,9].

Admission glucose is an attractive early marker because clinicians routinely measure it before substantial inpatient treatment. Rather than indicating diabetes, hyperglycemia during acute illness is often triggered by a combination of catecholamines, cortisol, glucagon, inflammatory signaling, and acute insulin resistance, which can produce stress hyperglycemia [10,11,12,13]. Thus, elevated glucose may therefore reflect systemic physiologic stress rather than directly cause renal dysfunction [14,15]. Acute hyperglycemia may therefore reflect several aspects of the patient’s response to decompensation, including neuroendocrine activation, altered substrate utilization, and insulin resistance. However, a single random blood sugar (RBS) measurement cannot distinguish chronic dysglycemia from an acute stress response, particularly when pre-existing diabetes and HbA1c data are unavailable or incomplete. Therefore, its clinical interpretation should account for the patient’s underlying glycemic status and the severity of the acute illness [16].

Observational studies have linked admission hyperglycemia to adverse outcomes in acute heart failure, including mortality [17,18,19]. However, a prognostic association with mortality does not equate to identifying CRS1 [20]. While a single random glucose measurement captures a combination of chronic and acute glycemic status, incorporating HbA1c and relative stress–hyperglycemia metrics may offer clinical context [21,22,23,24]. Recent studies have examined the stress–hyperglycemia ratio (SHR), which relates admission glucose to estimated average glucose derived from HbA1c, to distinguish acute glycemic elevation from chronic glycemic exposure. Studies in ADHF have reported associations between SHR and mortality or rehospitalization, although the direction and magnitude of these associations may vary by diabetes status and the population studied [15,16]. Evidence linking glycemic measures specifically to CRS1 continues to evolve. A recent acute heart failure cohort study evaluated CRS1 in relation to broader glycemic phenotypes defined by diabetes history, fasting glucose, and HbA1c, highlighting the relevance of glycemic status to cardiorenal risk assessment [25].

The present study addresses this gap by examining admission RBS in relation to CRS1 in a previously described Saudi ADHF cohort [26]. The primary objective was to determine whether admission RBS was independently associated with CRS1 in patients with ADHF. Secondary objectives were to compare RBS between CRS1 and non-CRS1 patients, assess its relationship with renal function, evaluate effect modification by diabetes, and explore hospitalization duration. Unlike studies focused primarily on mortality or composite cardiovascular outcomes, this analysis specifically evaluates the association between routinely measured admission RBS and CRS1 status at admission. CRS1 was classified at admission using clinical and renal information documented in the source dataset; the study therefore assesses concurrent group differences and associations rather than predicting incident CRS1 following glucose measurement. By examining adjusted associations and discriminatory performance in a Saudi cohort, the study aims to clarify the clinical relevance and limitations of admission RBS as a readily available marker in patients hospitalized with ADHF.

## 2. Materials and Methods

### 2.1. Study Design and Population Data Source

This was a retrospective secondary analysis of a previously described Saudi cohort of patients hospitalized with ADHF [26]. The present manuscript focuses exclusively on admission glycemia and CRS1 classification. CRS1 was defined as acute kidney injury (AKI) occurring in the setting of acute cardiac decompensation, per the Kidney Disease Improving Global Outcomes (KDIGO) criteria. KDIGO criteria define AKI as an increase in serum creatinine of ≥0.3 mg/dL within 48 h, an increase to ≥1.5 times baseline within 7 days, or urine output of <0.5 mL/kg/h for at least 6 h [27]. However, the retrospective dataset did not permit independent verification of all KDIGO criteria for every patient; therefore, CRS1 status was based on the classification recorded in the source dataset. RBS was analyzed in relation to this documented classification. In the parent cohort from the same institution, CRS1 was identified in approximately half of ADHF admissions, supporting the characterization of the present population as a CRS-enriched cohort. Detailed epidemiologic findings and CRS1-related analyses have been reported separately [26].

CRS1 status was established at admission based on the clinical and renal criteria documented in the original clinical dataset. Because CRS1 was identified at admission, this study examined the association between admission RBS and admission CRS1 status. It did not assess whether elevated glucose preceded or predicted subsequent CRS1 development, nor could it establish a causal relationship between admission glycemia and renal dysfunction. The retrospective analysis used the CRS1 classification available in the source records. In routine clinical practice, patients identified as having CRS1 with AKI underwent nephrology consultation for further evaluation and management. Nephrology consultation was part of the clinical care pathway and was not used as a criterion for defining CRS1 status in the study.

### 2.2. Study Population and Outcome

**Figure 1:**
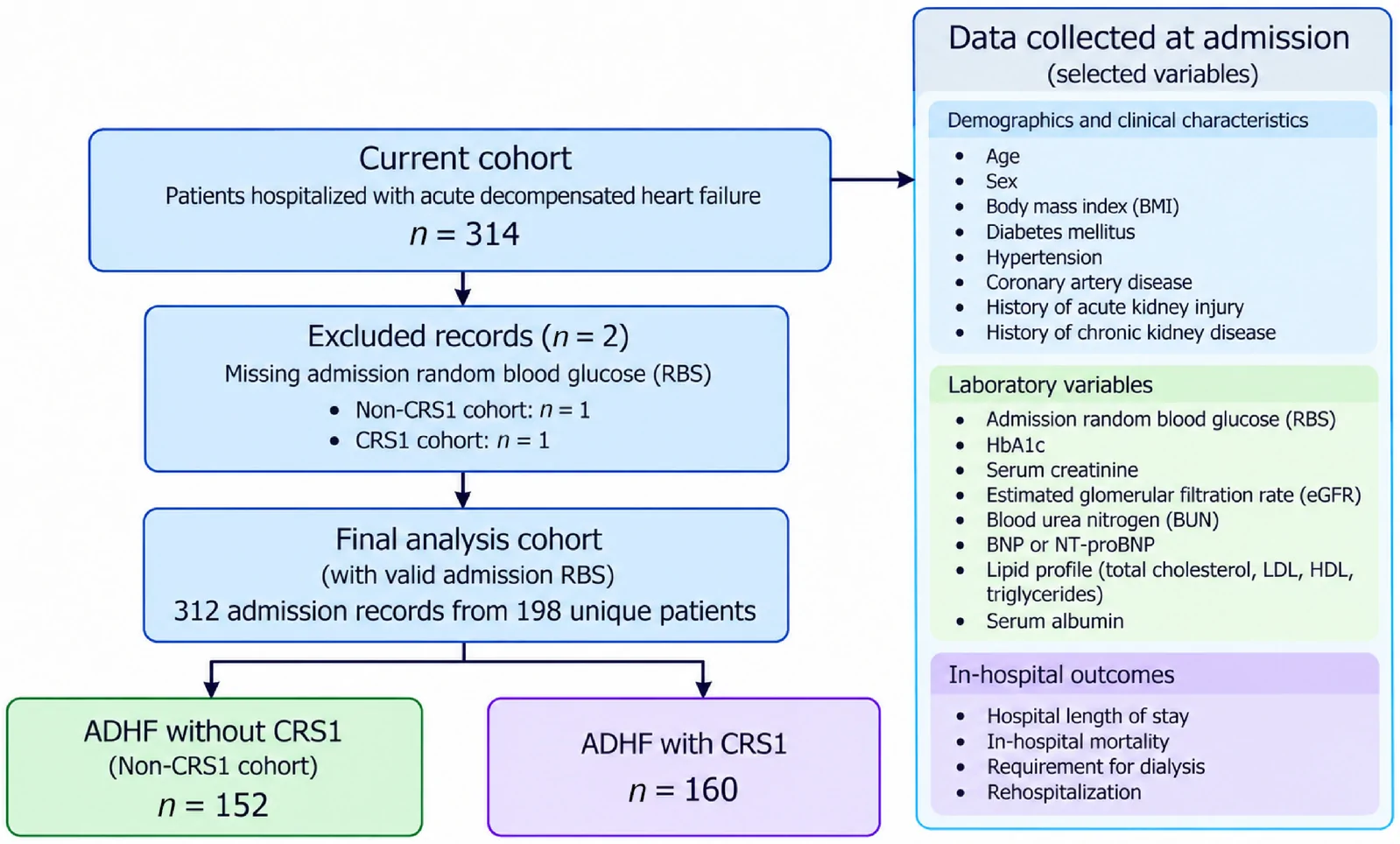
Study design and patient flow. The initial cohort comprised 314 admission records from patients hospitalized with acute decompensated heart failure (ADHF). Two records were excluded because of missing admission random blood sugar (RBS) values (one from each group). The final analysis included 312 admission records from 198 unique patients, comprising 152 non-cardiorenal syndrome type 1 (non-CRS1) records and 160 CRS1 records. The figure also summarizes the demographic, clinical, and laboratory variables collected at admission, along with selected in-hospital outcomes. HbA1c, glycated hemoglobin; eGFR, estimated glomerular filtration rate; BNP, B-type natriuretic peptide; NT-proBNP, N-terminal pro-B-type natriuretic peptide.

The source dataset contained 314 records: 153 non-CRS1 and 161 CRS1. Of these records, 2 patients were excluded because RBS was missing (Figure 1). CRS1 status was based on the classification recorded in the source dataset. Because the available records did not provide sufficient longitudinal information to independently verify the temporal relationship between acute cardiac deterioration and acute kidney injury, CRS1 status was not independently re-adjudicated using KDIGO criteria. Accordingly, the present analysis evaluated the association between admission RBS and the source-defined CRS1 classification.

### 2.3. Exposure and Covariates

The primary exposure was admission random blood sugar (RBS), measured in mmol/L. Prespecified covariates were age, sex, diabetes, hypertension, prior AKI, CKD, and COPD. eGFR and HbA1c were examined in sensitivity models. Admission RBS was categorized exploratorily as <7.8 mmol/L (normal range), 7.8–<11.1 mmol/L (prediabetes/borderline), and ≥11.1 mmol/L (diabetes range).

### 2.4. Statistical Analysis

We conducted statistical analyses using IBM SPSS Statistics (version 20.0; Armonk, NY, USA, IBM Corp., 2011). Continuous variables are presented as mean ± SD and categorical variables as *n* (%). RBS differences among groups were evaluated with Welch’s *t*-test and Mann–Whitney U test. Logistic regression modeled CRS1 per 1 mmol/L increase in RBS. Models were unadjusted; adjusted for age, sex, and diabetes; and further adjusted for hypertension, prior AKI, CKD, and COPD. Sensitivity models added eGFR or HbA1c. Interaction between diabetes and continuous RBS was tested.

Because multiple admission records could belong to the same patient, logistic regression models used cluster-robust standard errors based on the reconciled patient identifier to account for within-patient dependence. The models evaluated the association between RBS and CRS1 status, with sequential adjustment for demographic and clinical covariates. The number of admission records and unique patient clusters varied across models according to the availability of the variables included in each model. This clustering adjustment was applied to the regression models and not to the conventional between-group comparisons.

The discriminatory ability of admission RBS for CRS1 was assessed using receiver operating characteristic (ROC) analysis, with the area under the curve (AUC) used to quantify discrimination. To evaluate uncertainty while accounting for repeated admissions, a patient-level bootstrap sensitivity analysis was performed using 3000 resamples. In each resample, unique patients were sampled with replacement, and all eligible admission records belonging to each selected patient were retained. The AUC and its 95% bootstrap confidence interval were then estimated from the resampled data. The patient-level bootstrap was performed separately from the standard ROC analysis and from the cluster-robust regression models.

Hospital length of stay (LOS) was examined as an exploratory downstream outcome and categorized as ≤7 versus >7 days, but it was not treated as a predictor of CRS1. For exploratory sensitivity analysis, admission RBS was categorized using prespecified glucose cut points of <7.8, 7.8–<11.1, and ≥11.1 mmol/L. These categories were not intended as diagnostic thresholds for CRS1. All tests were two-sided, and *p* < 0.05 was considered statistically significant.

### 2.5. Ethical Considerations

This retrospective analysis evaluated historical data on patients with CRS1 admitted to the cardiology department who received a nephrology consultation between 1 April 2018, and 31 March 2023. The study relied solely on an existing dataset, with no active patient contact, clinical interventions, or treatment modifications. Ethical approval for the parent study was granted by the King Abdullah International Medical Research Center Institutional Review Board (NRA23A/032/07), with strict protocols to ensure complete data anonymization and confidentiality [26]. The requirement for informed consent was waived because of the retrospective design and use of de-identified data.

The authors acknowledge using ChatGPT (GPT-4o, OpenAI, San Francisco, CA, USA; https://chatgpt.com; accessed on 10 September 2026) for language editing, data cleaning, an initial literature review, and manuscript restructuring. The authors developed and verified all scientific content, data analysis, and interpretation. The authors assume full responsibility for the originality, validity, and overall integrity of the final version.

## 3. Results

### 3.1. Admission Characteristics

The analysis included 314 records: 153 non-CRS1 and 161 CRS1. Admission RBS was available for 152 non-CRS1 and 160 CRS1 records. Age and BMI were similar, whereas eGFR and several comorbidities differed between groups. Table 1 shows study admission characteristics.

### 3.2. Admission RBS and CRS1

**Figure 2:**
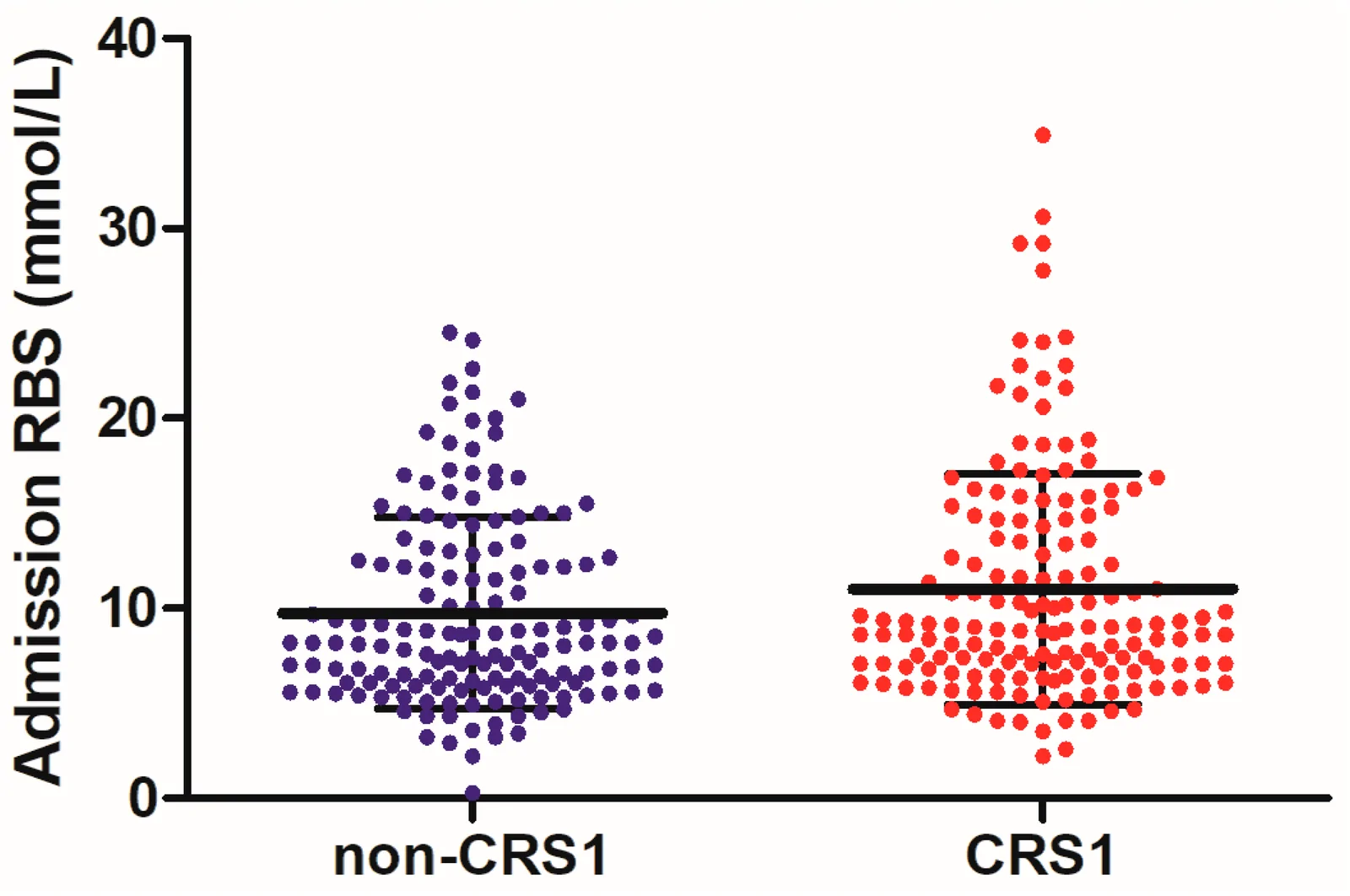
Admission random blood sugar in non-CRS1 and CRS1 records. Individual admission RBS values are shown as scatter points, with the mean ± SD indicated for each group. The mean admission RBS was higher in CRS1 than in non-CRS1 records (11.02 ± 6.07 vs. 9.74 ± 5.04 mmol/L; Welch’s *t*-test, *p* = 0.044). The corresponding Mann–Whitney U test showed a similar borderline association (*p* = 0.049).

Admission RBS was higher in CRS1 than in non-CRS1 records (mean ± SD: 11.02 ± 6.07 vs. 9.74 ± 5.04 mmol/L), with a mean difference of 1.27 mmol/L. Welch’s *t*-test (*p* = 0.044) and Mann–Whitney test (*p* = 0.049; Figure 2) showed a significant difference. RBS differed modestly between groups, but this difference did not persist as an independent association after adjustment (Table 2).

**Figure 3:**
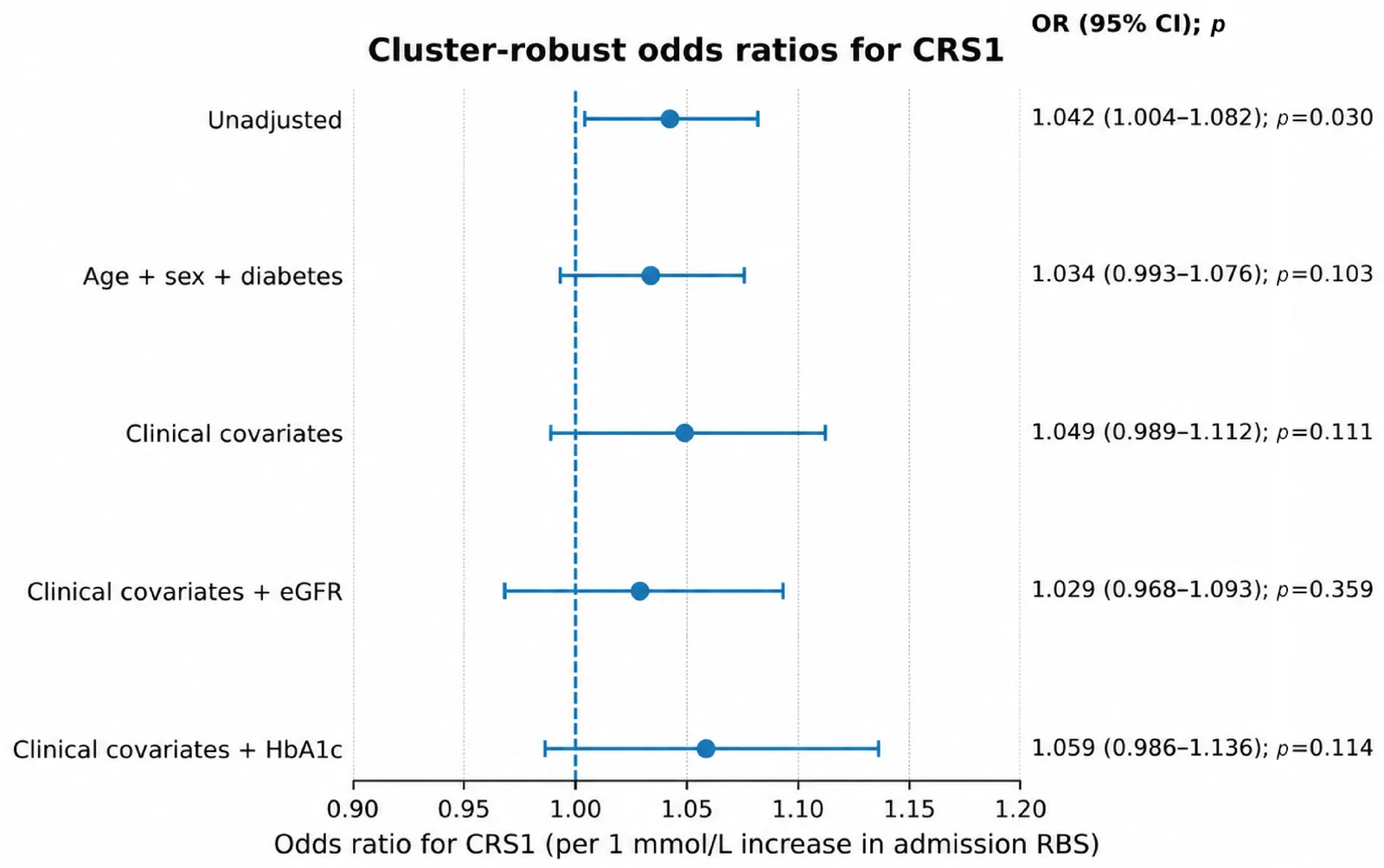
Cluster-robust odds ratios for CRS1 according to admission RBS. Odds ratios (OR) quantify the association between admission RBS and CRS1 for each 1 mmol/L increase in RBS. Models were estimated using logistic regression with standard errors clustered by medical record number. Sequentially adjusted models included age, sex, diabetes, hypertension, prior acute kidney injury, chronic kidney disease, and chronic obstructive pulmonary disease, and sensitivity models evaluated eGFR and HbA1c. *p*-values are reported for each model. Horizontal bars represent 95% confidence intervals, and the vertical line at OR = 1 indicates the null association.

In cluster-robust logistic regression, each 1 mmol/L increase in admission RBS was associated with higher odds of CRS1 in the unadjusted model (OR = 1.042, 95% CI = 1.004–1.082; *p* = 0.030; Table 2 and Figure 3). After adjusting for age, sex, and diabetes, the OR changed minimally, but the association was no longer statistically significant (OR = 1.034, 95% CI = 0.993–1.076; *p* = 0.103). In the expanded clinical model, the association remained positive but non-significant (OR = 1.049, 95% CI = 0.989–1.112; *p* = 0.111). Adding eGFR (OR = 1.029, 95% CI = 0.968–1.093; *p* = 0.359) or HbA1c (OR = 1.059, 95% CI = 0.986–1.136; *p* = 0.114) did not materially change the inference (Table 2 and Figure 3).

### 3.3. Categorized RBS and Discrimination

For exploratory categorical analysis, admission RBS was grouped into low (<7.8 mmol/L), intermediate (7.8–<11.1 mmol/L), and high (≥11.1 mmol/L) categories. These categories were used to assess the association between RBS group and CRS1 status and were not intended to establish clinical or diagnostic thresholds. Cluster-robust logistic regression results are shown in Table 3.

**Figure 4:**
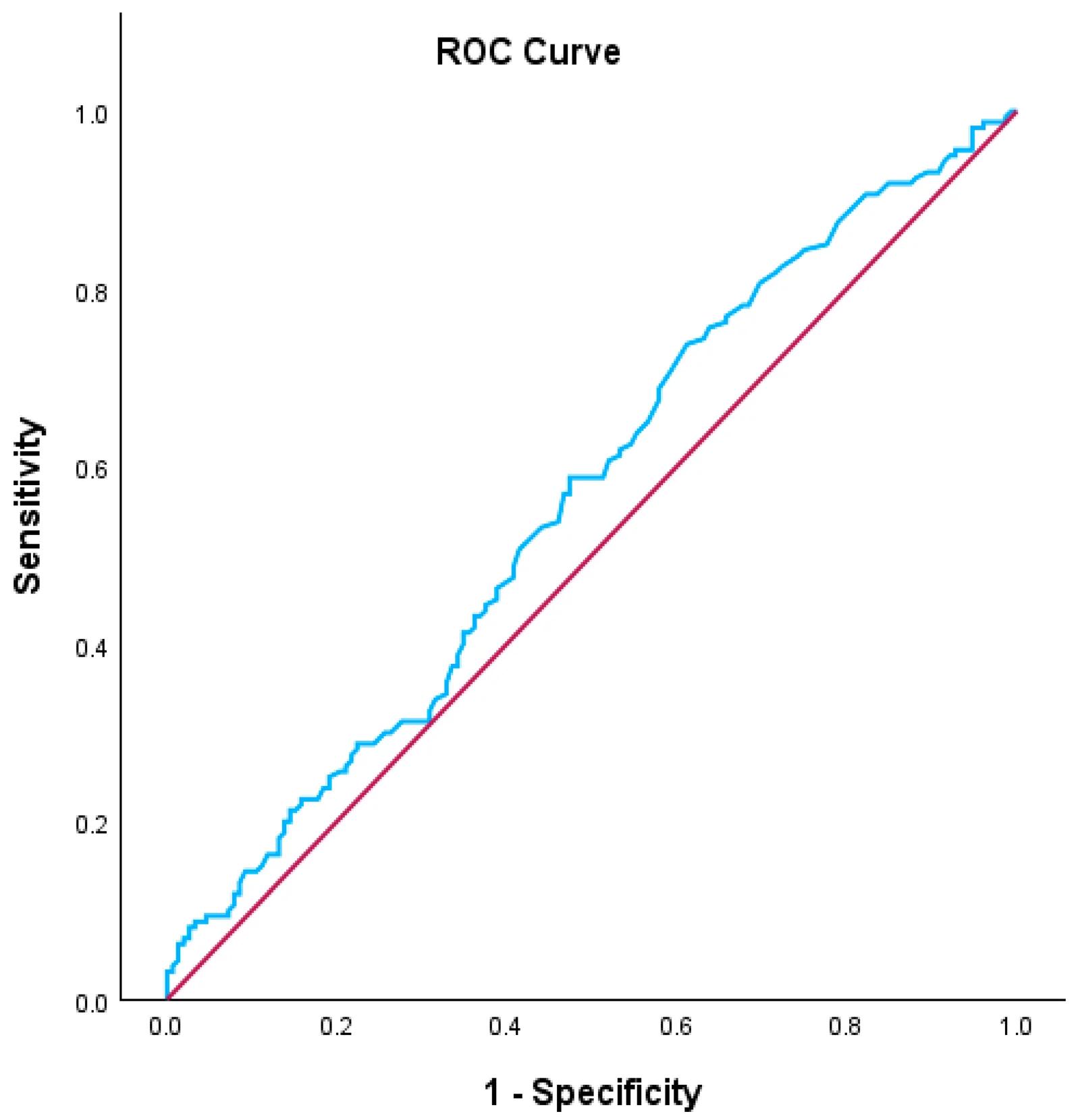
Receiver operating characteristic (ROC) curve for admission random blood sugar (RBS) in discriminating CRS1 from non-CRS1 records. The area under the curve (AUC) was 0.564, with a patient-level bootstrap 95% confidence interval of 0.501–0.627 based on 3000 resamples of 198 unique patients. The diagonal line (in red) represents chance-level discrimination. The results indicate limited discriminatory ability of admission RBS for identifying cardiorenal syndrome type 1 (CRS1).

Using <7.8 mmol/L as the reference category, neither 7.8–<11.1 nor ≥11.1 mmol/L was significantly associated with CRS1 in unadjusted or adjusted analyses (Table 3). RBS categories were similar between groups (Table 4). Admission RBS demonstrated poor discrimination for CRS1 (AUC = 0.564, 95% bootstrap CI = 0.501–0.626, Figure 4).

### 3.4. Receiver Operating Characteristic Analysis and Sensitivity Analysis

Admission RBS showed limited discriminatory ability for identifying CRS1. The area under the receiver operating characteristic (ROC) curve was 0.564, with a 95% patient-level bootstrap confidence interval (CI) of 0.501–0.627 based on 3000 resamples of 198 unique patients. To assess the potential influence of repeated admissions, patient-level bootstrap resampling was performed while retaining all eligible admission records for each sampled patient. The resulting AUC and confidence interval indicated limited discrimination, suggesting that admission RBS alone has limited utility for distinguishing CRS1 from non-CRS1 records (Figure 4 and Table 5).

### 3.5. Association Between Admission RBS and Renal Function Among Non-CRS1s and CRS1 Patients

**Figure 5:**
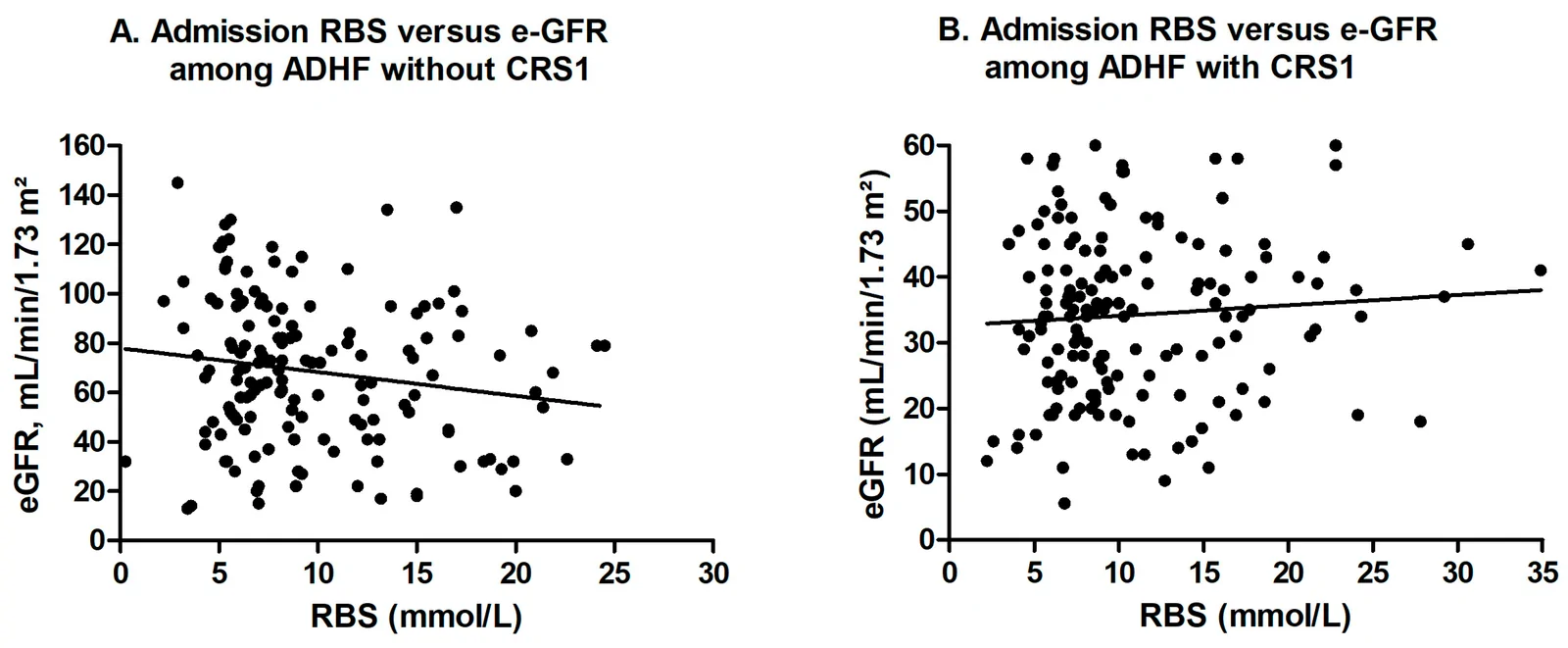
Association between admission RBS and eGFR according to CRS1 status. Scatterplots show the relationship between admission RBS and eGFR in (**A**) non-CRS1 records (*n* = 151) and (**B**) CRS1 records (*n* = 160). Spearman’s rank correlation showed a weak inverse association in the non-CRS1 group (ρ = −0.175, *p* = 0.032), whereas no statistically significant association was observed in the CRS1 group (ρ = 0.062, *p* = 0.435). eGFR represents renal function at the time of assessment; therefore, these cross-sectional correlations do not assess renal recovery or AKI severity.

In the non-CRS1 group, admission RBS showed a weak inverse correlation with eGFR (Spearman ρ = −0.175, *p* = 0.032; *n* = 151; Figure 5A). In contrast, no significant correlation was observed among patients with CRS1 (Spearman ρ = 0.062, *p* = 0.435; *n* = 160; Figure 5B). These findings do not support a meaningful relationship between admission glycemia and cross-sectional renal function among patients with CRS1.

### 3.6. Exploratory Hospitalization Length of Stay (LOS)

Hospitalization duration was interpretable in 152 non-CRS1 and 51 CRS1 records. Prolonged hospitalization (>7 days) occurred in 26/152 (17.1%) non-CRS1 and 10/51 (19.6%) CRS1 records. CRS1 was not associated with >7-day hospitalization in either the unadjusted or age-, sex-, and diabetes-adjusted models, and admission RBS was not associated with prolonged hospitalization (Table 6). Although admission RBS showed a weak positive correlation with continuous hospitalization duration (Spearman ρ = 0.160, *p* = 0.026; *n* = 192), this finding should be interpreted cautiously because LOS is downstream of admission status and duration data were incomplete.

LOS data were available for 203 of the 314 admission records, including 152/153 (99.3%) non-CRS1 records and 51/161 (31.7%) CRS1 records. Some CRS1 entries in the source dataset were labeled as “out-patient visit” rather than having a numerical hospitalization duration. These entries may partly explain the incomplete LOS data; however, the reasons for missingness could not be definitively established. Given the substantial difference in data availability between groups, LOS analyses were considered exploratory.

## 4. Discussion

The principal finding was that admission RBS was higher among records classified as CRS1 than among those without CRS1. However, this association was attenuated after adjustment for clinical risk factors and was no longer statistically significant. Thus, although higher admission glycemia was associated with CRS1 at the unadjusted level, the relationship was not independent of the patients’ broader clinical characteristics. The limited discriminatory performance of admission RBS further argues against its use as a stand-alone marker for CRS1. Collectively, these findings suggest that the clinical value of admission RBS may lie more in characterizing the metabolic state of patients presenting with ADHF than in independently identifying CRS1.

An important consideration in interpreting these findings is that CRS1 status was established at admission, rather than identified as a new event after the admission glucose measurement. Therefore, the present analysis evaluates the association between admission RBS and CRS1 status at admission, rather than RBS’s ability to predict subsequent CRS1 development. Although admission RBS may reflect the metabolic state at presentation, these data do not establish whether hyperglycemia preceded renal dysfunction or whether elevated glucose contributed to the development of CRS1. Accordingly, the findings should be interpreted as evidence of an association with the admission CRS1 phenotype rather than as evidence of causation or prospective risk prediction.

Attenuation after adjustment suggests that admission hyperglycemia may partly reflect shared vulnerability and acute illness severity rather than independently determining CRS1. Acute cardiac decompensation involves counter-regulatory hormonal and neurohormonal activation, insulin resistance, inflammation, and renal hemodynamic stress, while renal venous congestion and impaired effective perfusion may further contribute to kidney dysfunction [3,4,5,6]. Conversely, catecholamines, cortisol, glucagon, inflammatory signaling, and acute insulin resistance can increase blood glucose during acute illness [10,11,12,13]. A single admission glucose value may therefore reflect a mixture of chronic glycemic status and acute metabolic stress. Cox et al. support this interpretation, finding that admission glucose in acute heart failure often closely matches chronic glycemic status, whereas a marked increase above chronic glycemia was more strongly associated with adverse outcomes [26]. These findings support the possibility that absolute admission glucose integrates information on pre-existing dysglycemia and the acute physiological response to decompensation, neither of which a single RBS measurement can distinguish.

Our findings are broadly consistent with the broader acute-heart-failure literature, in which admission hyperglycemia has been linked to mortality and other adverse outcomes [17,18,19,21]. However, these prognostic associations should not be equated with CRS1 identification. This distinction matters because CRS1 is biologically driven by renal vulnerability, congestion, hemodynamic alterations, and neurohormonal and inflammatory pathways. These factors may coexist with hyperglycemia but are not necessarily determined by admission glycemia [3,4,5,6]. In a separate acute-heart-failure cohort, Fan et al. found that fasting blood glucose was associated with CRS1 in univariate analysis but was not retained as an independent predictor in the final multivariable model, which included age, diabetes, NYHA class, eGFR, hs-CRP, and urinary angiotensinogen [28]. This finding is consistent with our finding that admission RBS was associated with CRS1 before adjustment but not after accounting for clinical and renal characteristics. Differences in glucose sampling conditions, patient characteristics, renal function, and covariate selection may partly explain variation across studies. Importantly, fasting glucose and admission RBS are not interchangeable, and findings from one measurement strategy should not be generalized to another.

The distinction between absolute glucose and stress-related glycemic elevation also warrants consideration. A random glucose measurement does not account for the patient’s prior glycemic exposure and may therefore carry different implications for patients with and without established diabetes. Researchers have investigated the stress–hyperglycemia ratio (SHR), calculated from admission glucose and estimated average glucose derived from HbA1c, as a complementary measure of acute glycemic elevation relative to chronic glycemia. A meta-analysis of 10 heart-failure studies involving 15,250 patients found that higher SHR was associated with mortality and other adverse cardiovascular outcomes, although substantial heterogeneity was observed for some outcomes [29]. These findings support further investigation of relative stress–hyperglycemia measures in acute heart failure, but they do not establish that SHR improves CRS1 classification or that it is superior to admission RBS for identifying CRS1.

Because HbA1c was unavailable for some patients in the present cohort, this distinction could not be examined comprehensively. The relationship between admission glycemia and hospitalization duration has also been inconsistent across studies. In patients with diabetes and acute heart-failure exacerbation, Bhatia et al. reported a positive correlation between admission glucose and hospitalization duration, while Gebreegziabher et al. similarly found that admission glucose correlated with length of stay after adjustment for age, sex, weight, hypertension, renal function, and anemia [30,31]. In contrast, Dhand et al. found no statistically significant difference in hospitalization duration across fasting glucose categories in patients with congestive heart failure, although the small subgroup with higher glucose had numerically longer stays [32]. These divergent findings suggest that the relationship between glycemia and LOS may depend on one or more factors, including diabetes status, chronic glycemic control, renal function, illness severity, glucose definition, and the clinical processes determining discharge. Differences in study design and LOS categorization may also limit direct comparisons between cohorts. Consequently, the available evidence does not support a consistent relationship between a single admission glucose measurement and hospitalization duration across acute heart failure populations.

Our exploratory LOS findings therefore warrant a more cautious interpretation. Admission RBS showed only a weak positive correlation with continuous hospitalization duration, and neither CRS1 status nor admission RBS was significantly associated with hospitalization longer than 7 days. The weak RBS-LOS correlation should not be interpreted as evidence that admission glycemia drives prolonged hospitalization, because LOS is a downstream outcome that could be influenced by CRS1, treatment response, complications, comorbidity, and discharge practices [33,34]. In addition, hospitalization duration was incompletely recorded in our cohort, particularly in CRS1 records, substantially reducing the available sample for this analysis. Accordingly, our LOS analysis should be considered exclusively exploratory and should not be used to infer causality. Future analyses should use more complete hospitalization data and account for clinical severity, renal outcomes, treatment courses, and discharge-related factors before drawing conclusions about the relationship between glycemia and resource utilization.

The present findings should also be interpreted in relation to the previously published report from this Saudi cohort [26,35] by examining admission glycemia in relation to CRS1 status and hospitalization duration. It addresses a distinct question and does not assume that admission glycemia explains CRS1 development or the hospitalization burden associated with this condition. The present analysis therefore complements the earlier cohort report by examining admission glycemia as a potential clinical marker, without assuming that it explains CRS1 development or the increased hospitalization burden associated with this condition.

Taken together, these findings suggest that admission glycemia may reflect the metabolic stress of acute decompensation, whereas underlying renal vulnerability, congestion, hemodynamic burden, comorbidity profile, and subsequent clinical course may be more directly related to the multifactorial pathophysiology of CRS1 and its clinical development. Admission glycemia may therefore provide contextual information within a high-risk ADHF phenotype, but it is unlikely to be sufficient for CRS1 classification on its own. Future risk-stratification approaches may benefit from integrating metabolic stress with congestion, renal vulnerability, hemodynamics, serial renal function, and validated kidney-injury biomarkers. However, prospective validation is needed to confirm the incremental value of these combined measures.

The association between admission RBS and eGFR was weak and differed by CRS1 status, with a significant inverse correlation observed only in the non-CRS1 group. Given the cross-sectional design, these findings cannot establish a causal relationship between glycemia and renal dysfunction or provide evidence of renal recovery or AKI severity. Moreover, eGFR reflects renal function at a single time point, whereas CRS1 involves temporal changes in cardiac and renal function; therefore, the absence of a meaningful correlation in the CRS1 group should be interpreted cautiously. Serial creatinine, urine output, congestion measures, and kidney-injury biomarkers would better address the underlying biology [27,30,36,37]. No evidence showed that diabetes modified the RBS–CRS1 association, although the smaller non-diabetic subgroup limits power for interaction testing. Future studies should incorporate HbA1c and relative stress–hyperglycemia measures to distinguish chronic glycemia from acute metabolic perturbation [19,22]. From a translational perspective, these observations do not justify empiric glucose-lowering solely to prevent CRS1. Instead, they support evaluating whether more comprehensive glycemic phenotyping can improve clinical assessment when interpreted alongside established indicators of cardiorenal severity.

Prospective studies should determine whether stress–hyperglycemia measures or longitudinal glycemic trajectories provide incremental predictive value beyond established clinical, congestion, hemodynamic, and renal variables, and whether metabolic phenotyping can identify patients who would benefit from targeted intervention.

## 5. Strengths and Limitations

This study addresses a focused clinical question using routinely available admission RBS data. It evaluates RBS both continuously and categorically, applies sequential multivariable adjustment, and incorporates complementary analyses of interaction, discrimination, renal function, and hospitalization. This retrospective secondary analysis limits causal inference and remains susceptible to residual confounding. Admission RBS was based on a single measurement and could not distinguish chronic dysglycemia from acute stress hyperglycemia. Moreover, food intake and pre-measurement treatment may have influenced glucose levels. HbA1c was unavailable for some records, reducing the sample available for sensitivity analysis. Another limitation is that CRS1 status was retained from the source dataset and could not be independently re-adjudicated using complete longitudinal clinical data. In particular, the available data were insufficient to verify consistently the temporal sequence of acute cardiac deterioration and AKI or to independently assess all cases against KDIGO criteria using baseline and serial serum creatinine measurements and urine-output data. The study also did not establish AKI severity or renal recovery, which would require appropriately timed serial renal measurements and additional clinical information. Consequently, we cannot rule out potential outcome misclassification, and the findings should be interpreted as associations with recorded CRS1 status rather than evidence of the temporal development of CRS1 following admission RBS measurement.

Of particular note, substantial and unequal data incompleteness limited LOS analyses, with only 31.7% of CRS1 records having available LOS data, compared with 99.3% of non-CRS1 records. Although some CRS1 entries were labeled as outpatient visits, the reasons for missing LOS data could not be confirmed. This imbalance may have introduced selection bias and limits the interpretation and generalizability of the LOS findings. Accordingly, the absence of statistically significant associations should not be interpreted as evidence that no association exists. Additionally, the glucose categories were exploratory and should not be interpreted as validated diagnostic thresholds for CRS1. Finally, the ROC analysis demonstrated limited discrimination and should not be used to derive a clinical RBS cutoff for identifying CRS1. Furthermore, because CRS1 was classified at admission and the available retrospective data could not establish the temporal relationship between the onset of hyperglycemia and renal dysfunction, the direction of the observed association remains uncertain. Therefore, single admission RBS measurement should not be interpreted as a predictor of incident CRS1 or as evidence of a causal relationship. Prospective studies with standardized baseline renal function assessment, serial glucose measurements, and clearly defined AKI onset timing are needed to determine whether admission glycemia adds prognostic information for subsequent cardiorenal complications.

## 6. Conclusions

In conclusion, admission RBS was higher in patients with CRS1 and was associated with CRS1 in unadjusted analysis; however, this association weakened after adjustment, and RBS alone had limited discriminatory ability. These findings suggest that admission glycemia may reflect metabolic stress in patients with ADHF and CRS1 rather than serve as an independent or stand-alone classifier of CRS1. The exploratory length-of-stay analysis did not establish a significant association between prolonged hospitalization and either CRS1 or admission RBS. Prospective studies incorporating serial glucose measurements, HbA1c, stress–hyperglycemia indices, and integrated assessments of congestion, hemodynamics, and renal biomarkers are warranted to determine whether these measures can add value to early CRS1 risk stratification and clinical decision-making.

## Figures and Tables

**Table 1:** Cohort admission characteristics.

Variable	Non-CRS1	CRS1	*p* Value
Demographics			
Age, years	67.30 ± 12.36	68.33 ± 12.54	0.469
BMI, kg/m^2^	32.87 ± 8.71	32.08 ± 8.40	0.414
Male sex, *n* (%)	78 (51.0%)	90 (55.9%)	0.447
Comorbidities			
Diabetes mellitus, *n* (%)	123 (80.4%)	139 (86.3%)	0.206
Hypertension, *n* (%)	131 (85.6%)	156 (96.9%)	<0.001
Chronic kidney disease, *n* (%)	45 (29.4%)	101 (62.7%)	<0.001
History of AKI, *n* (%)	16 (10.5%)	102 (63.4%)	<0.001
COPD, *n* (%)	8 (5.2%)	22 (13.7%)	0.019
Admission laboratory variables			
Heart rate, beats/min	87.73 ± 15.46	80.48 ± 13.25	<0.001
HbA1c, %	7.61 ± 1.89	7.65 ± 2.02	0.855
Admission RBS, mmol/L	9.74 ± 5.04	11.02 ± 6.07	0.044
eGFR, mL/min/1.73 m^2^	68.71 ± 29.77	34.20 ± 12.59	<0.001
Creatinine, µmol/L	109.97 ± 59.25	186.17 ± 78.67	<0.001
BUN, mmol/L	9.18 ± 5.44	16.12 ± 7.46	<0.001

Data are presented as mean ± SD or *n* (%). Continuous variables were compared using Welch’s *t*-test and categorical variables using chi-square tests. Length of stay is a downstream hospitalization outcome and is included for descriptive completeness. LOS was analyzed only when a day-based entry was interpretable. *p* < 0.05 was considered statistically significant.

**Table 2:** Cluster-robust logistic regression of continuous admission RBS and CRS1.

Model	*n*	Unique Patients Clusters	OR per 1 mmol/L	95% CI	*p*
Unadjusted	312	198	1.042	1.004–1.082	0.030
Age + sex + diabetes	307	194	1.034	0.993–1.076	0.103
Clinical covariates *	307	194	1.049	0.989–1.112	0.111
Clinical covariates + eGFR	306	194	1.029	0.968–1.093	0.359
Clinical covariates + HbA1c	287	184	1.059	0.986–1.136	0.114

* Clinical covariates: age, sex, diabetes, hypertension, prior AKI, CKD, and COPD. The analytical unit was the admission record. Because some patients contributed more than one record, the primary continuous-RBS clinical model included 307 records from 194 patients. Cluster-robust standard errors were used to account for multiple admission records from the same patient. Differences in sample size across models reflect the availability of covariates required for each model. The RBS categories were used for exploratory analysis and should not be interpreted as diagnostic thresholds for CRS1. Because renal dysfunction is central to CRS1 classification, we treated the eGFR-adjusted model as a sensitivity analysis rather than the primary model. In contrast, the HbA1c-adjusted model used a smaller complete-case sample.

**Table 3:** Cluster-robust logistic regression using categorized admission RBS.

Model	Admission RBS Category	*n*	Unique Patient Clusters	OR	95% CI	*p* Value
Model 1: Unadjusted	<7.8 mmol/L (Reference)	312	198	1.000	Reference	-
Model 1: Unadjusted	7.8–<11.1 mmol/L	312	198	1.533	0.888–2.647	0.125
Model 1: Unadjusted	≥11.1 mmol/L	312	198	1.288	0.754–2.201	0.354
Model 2: Adjusted for age, sex, and diabetes	<7.8 mmol/L (Reference)	307	194	1.000	Reference	-
Model 2: Adjusted for age, sex, and diabetes	7.8–<11.1 mmol/L	307	194	1.307	0.738–2.315	0.358
Model 2: Adjusted for age, sex, and diabetes	≥11.1 mmol/L	307	194	1.123	0.637–1.981	0.688
Model 3: Adjusted for age, sex, diabetes, hypertension, and COPD	<7.8 mmol/L (Reference)	307	194	1.000	Reference	-
Model 3: Adjusted for age, sex, diabetes, hypertension, and COPD	7.8–<11.1 mmol/L	307	194	1.165	0.628–2.158	0.628
Model 3: Adjusted for age, sex, diabetes, hypertension, and COPD	≥11.1 mmol/L	307	194	1.116	0.616–2.024	0.717

Results are presented as odds ratios (ORs) with 95% confidence intervals (CIs), using RBS < 7.8 mmol/L as the reference category. Model 1 was unadjusted; Model 2 was adjusted for age, sex, and diabetes; and Model 3 was adjusted for age, sex, diabetes, hypertension, and COPD. Cluster-robust standard errors accounted for repeated admissions within patients. Model 1 included 312 admission records from 198 unique patient clusters; Models 2 and 3 included 307 records from 194 clusters each because of covariate availability. RBS categories were exploratory and should not be interpreted as validated diagnostic thresholds for CRS1. The reference category (Reference) was assigned an OR of 1.000, and all other ORs represent comparisons with this category.

**Table 4:** Distribution of admission RBS categories and association with CRS1.

Admission RBS Category	Non-CRS1, *n* (%)	CRS1, *n* (%)	Overall	*p*-Value
Low RBS category: <7.8 mmol/L	69 (45.4%)	60 (37.5%)	129 (41.3%)	
Intermediate RBS category: 7.8–<11.1 mmol/L	33 (21.7%)	44 (27.5%)	77 (24.7%)	
High RBS category: ≥11.1 mmol/L	50 (32.9%)	56 (35.0%)	106 (34.0%)	
Overall	152 (100%)	160 (100%)	312 (100%)	0.311

Admission RBS was categorized into low (<7.8 mmol/L), intermediate (7.8–<11.1 mmol/L), and high RBS (≥11.1 mmol/L). These categories were used for exploratory analysis and are not intended as diagnostic cutoffs for CRS1. The overall *p*-value is from chi-square testing across the three RBS categories.

**Table 5:** ROC analysis and patient-level bootstrap sensitivity analysis.

Analysis	Resampling Unit	Records (*n*)	Unique Patients (*n*)	Resamples	AUC	95% Bootstrap CI
Primary ROC analysis	Admission records	312	198	3000	0.564	0.501–0.627
Sensitivity analysis	Unique patients, retaining all eligible records per sampled patient	312	198	3000	0.564	0.501–0.627

The analysis included 312 admission records from 198 unique patients. The primary ROC analysis assessed the discriminatory ability of admission RBS for CRS1. The patient-level bootstrap sensitivity analysis sampled unique patients with replacement and retained all eligible admission records belonging to each selected patient. The patient-level bootstrap 95% CI was estimated from 3000 resamples. An AUC of 0.564 indicates limited discriminatory ability of admission RBS for identifying CRS1. Abbreviations: AUC, area under the receiver operating characteristic curve; CI, confidence interval; CRS1, cardiorenal syndrome type 1; RBS, random blood sugar.

**Table 6:** Exploratory hospitalization length of stay analysis using a 7-day cutoff.

Analysis	N	Estimate	95% CI	*p*
LOS: ≤7 vs. >7 days; CRS1 vs. non-CRS1	203	OR = 1.18	0.53–2.66	0.686
LOS: ≤7 vs. >7 days; CRS1, age + sex + diabetes adjusted	203	OR = 1.14	0.51–2.54	0.759
LOS: ≤7 vs. >7 days: admission RBS	192	OR = 1.10	0.92–1.31	0.306
Continuous LOS vs. admission RBS	192	Spearman ρ = 0.160	-	0.026
Continuous LOS, non-CRS1	152	5.61 ± 4.07 days	-	-
Continuous LOS, CRS1	51	7.08 ± 6.69 days	-	-

N represents the number of records with available data for the respective analysis. Continuous hospitalization length of stay (LOS) is presented as mean ± SD. Odds ratios (OR) were estimated using cluster-robust logistic regression with clustering by MRN. Regression analyses used 203 records for CRS1 comparisons and 192 records for analyses involving admission RBS. LOS data completeness was substantially imbalanced between groups: 99.3% in non-CRS1 versus 31.7% in CRS1. The CRS1 continuous LOS analysis included 51 records because only 51 of the 161 CRS1 records had interpretable hospitalization length-of-stay data. Abbreviations: LOS, length of stay; CRS1, cardiorenal syndrome type 1; RBS, random blood sugar; OR, odds ratio; CI, confidence interval; SD, standard deviation. LOS analyses should be interpreted as exploratory because of potential selection bias. Continuous LOS is presented as mean ± SD. The ORs represent the odds of LOS > 7 days relative to ≤7 days.

## Data Availability

The data are not publicly available due to institutional data governance policies at King Abdulaziz Hospital, MNGHA. De-identified data may be available from the corresponding author upon reasonable request and subject to institutional approval.

## Abbreviations

The following abbreviations are used in this manuscript:

ADHF	Acute decompensated heart failure
AKI	Acute kidney injury
AUC	Area under the curve
BMI	Body mass index
BNP	B-type natriuretic peptide
CAD	Coronary artery disease
CI	Confidence interval
COPD	Chronic obstructive pulmonary disease
CRS1	Cardiorenal syndrome type 1
CKD	Chronic kidney disease
eGFR	Estimated glomerular filtration rate
Hb	Hemoglobin
HbA1c	Glycated hemoglobin
HR	Heart rate
HTN	Hypertension
KDIGO	Kidney Disease: Improving Global Outcomes
LOS	Length of stay
MRN	Medical record number
NYHA	New York Heart Association
OR	Odds ratio
PVD	Peripheral vascular disease
RBS	Random blood sugar
ROC	Receiver operating characteristic
SD	Standard deviation
